# Influence of gender on diaphragm thickness using a method for determining intima media thickness in healthy young adults

**DOI:** 10.1186/s12880-022-00748-y

**Published:** 2022-02-11

**Authors:** Masayoshi Oguri, Tohru Okanishi, Takuya Ikeguchi, Kaoru Ogo, Sotaro Kanai, Yoshihiro Maegaki, Shinichi Wada, Takashi Himoto

**Affiliations:** 1 Department of Medical Technology, Kagawa Prefectural University of Health Sciences, 761-0123 Hara 281-1, Mure-cho, Takamatsu, Japan; 2 Division of Child Neurology, Institute of Neurological Sciences, Faculty of Medicine, Tottori University, Yonago, Japan; 3 Department of Clinical Laboratory, Tottori University Hospital, Yonago, Japan; 4 Department of Clinical Laboratory Science, Tenri Health Care University Faculty of Health Care, Tenri, Japan

**Keywords:** Diaphragm thickness, Ultrasonography, Respiratory muscle strength, Spine position

## Abstract

**Background:**

To clarify the differences in diaphragm thickness between male and female participants in healthy young adults with ultrasonography using the mean intima media thickness (IMT) method and to investigate the relationship between diaphragm thickness and respiratory pressure.

**Methods:**

Twenty-nine healthy individuals (16 females and 13 males) participated in the study. Diaphragm thickness was measured at total lung capacity (TLC) and at functional residual capacity (FRC) in each participant. We measured the diaphragm thickness using a method for mean intima media thickness. Moreover, change ratio of diaphragm thickness was calculated with the diaphragm thickness at TLC and FRC.

**Results:**

Mean diaphragm thicknesses at FRC in males were significantly narrower than those in females (p < 0.001). The change ratio of diaphragm thickness was significantly augmented in males compared with that in females (p < 0.001). There was a positive correlation between the change ratio of diaphragm thickness and pulmonary function data and respiratory muscle strength in healthy young adults.

**Conclusions:**

The change ratio of diaphragm thickness using the IMT method can be accurately performed with a high degree of reproducibility by clinical laboratory technicians and may be a useful indicator for evaluating diaphragm muscle strength.

## Background

Diaphragm movement and function can be assessed through various methods, including chest X-ray, CT, and MRI scans; pulmonary function test; evaluation of maximum respiratory pressure; and electromyography. Diaphragm ultrasonography is a cost-beneficial method that enables easy assessment of diaphragm thickness; moreover, it is a noninvasive bedside tool that can assess diaphragm function and movement [1]. At the bedside, we can measure diaphragm thickness using the B-mode with a linear probe, and diaphragm movement using M-mode with a convex probe, via ultrasonography [2, 3]. Recently, many reports have demonstrated associations among respiratory muscle strength, neuromuscular disorders, and chronic obstructive pulmonary disease assessed using ultrasonography [4, 5]. However, few studies have been published regarding the difference in diaphragm thickness between the sexes.

The respiratory pressure test is a useful method to assess respiratory muscle strength in clinical practice [6]. The evaluation of respiratory pressure is often used to determine muscle weakness in patients with muscular dystrophy, assess disease severity, and postulate prognosis [7]. Several studies were performed to define normative values for maximal respiratory pressure in children and adults, considering factors such as age, sex, height, and body weight [8, 9]; however, there are no reports regarding the relationship between diaphragm thickness with ultrasonography using the mean intima media thickness (IMT) method and maximal respiratory pressure in healthy young adults.

This study aimed to determine diaphragm thickness with ultrasonography using the IMT method in male and female healthy young adults. Additionally, we investigated the relationship between diaphragm thickness and maximal respiratory pressure.

## Methods

### Participants

This study was a prospective observational study conducted from October 2016 to March 2019. The study was approved by the Institutional Review Board of the Kagawa Prefectural University of Health Sciences, and informed consent was obtained from the participants before the study. Additionally, the study was conducted in accordance with the Declaration of Helsinki. First, 11 healthy participants (8 females and 3 males) aged 21–22 years were enrolled to investigate data on the intra-analyst and inter-analyst variabilities in order to evaluate the accuracy of the mean diaphragm thickness (MDT) using the IMT method. Subsequently, other 29 healthy participants (16 females, 13 males) aged 19–34 years were enrolled to evaluate the DMT thickness and relationship between diaphragm thickness and maximal respiratory pressure. The participants were either students or staff at Kagawa Prefectural University of Health Sciences and Tottori University. Body weight and height were measured, and The Du Bois and Du Bois body surface area (BSA) [10] and body mass index (BMI) were calculated for each participant. Participants with obesity (BMI > 25), neuromuscular disease, cardiorespiratory disease, or chronic illness were excluded.

### Observer training

The 1st and 2nd observers, who were students at Kagawa Prefectural University of Health Sciences and conducted the ultrasonography, were provided training ultrasonographic measurements of the diaphragm thickness by MO in 2 sessions, each session lasting 15 min.

### Ultrasound imaging and analysis

**Fig. 1:**
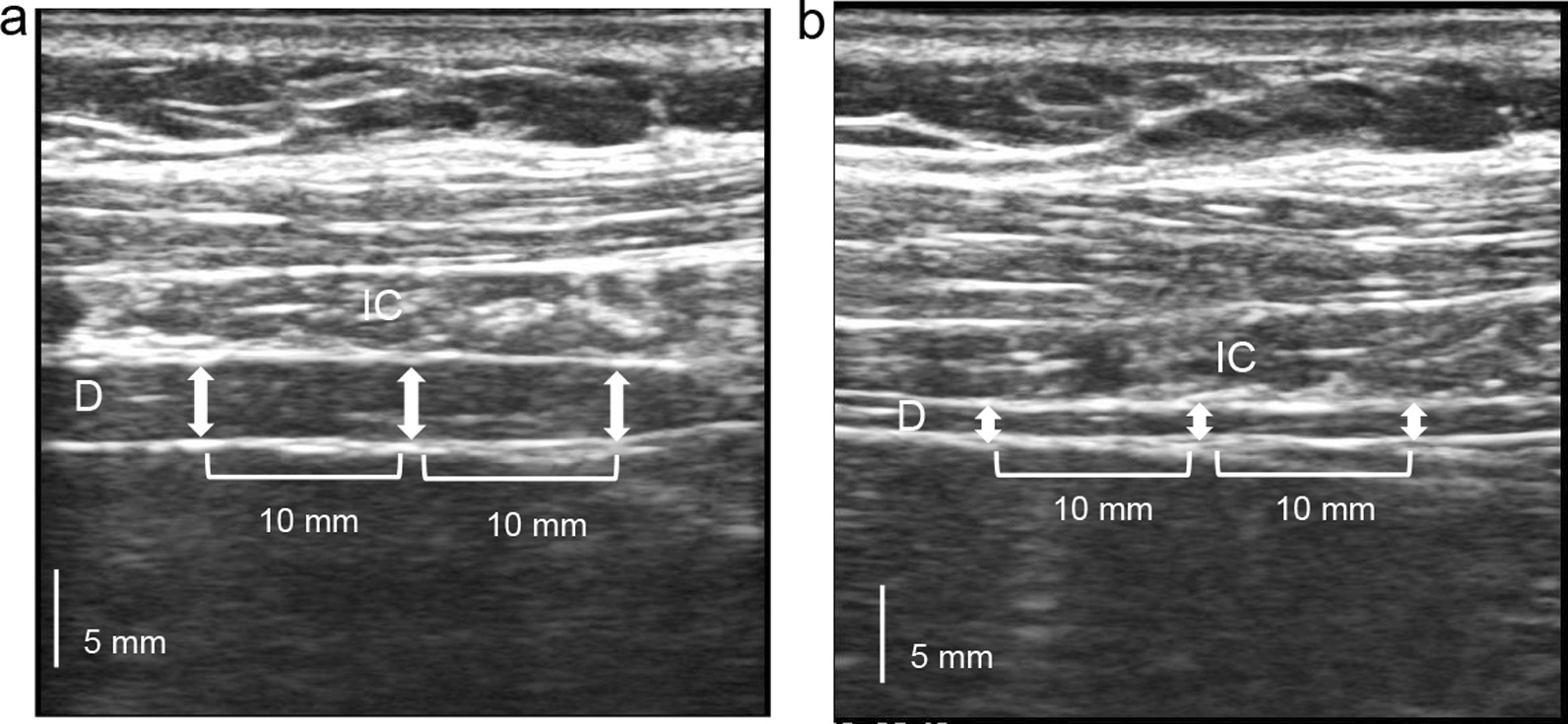
Representative ultrasound of the diaphragm. Diaphragm at total lung capacity (**a**) and functional residual capacity (**b**). The diaphragm (D, white bidirectional arrows) is the 3-layered structure situated deep to the intercostal (IC) muscles spanning two ribs; it is identified as the last set of parallel lines, with the pleural and peritoneal membranes overlying the less echogenic muscle. We calculated a mean of three points using the IMT software: the center of the diaphragm thickness, and two surrounding points on either side (each 10 mm from the center of the diaphragm thickness)

Using an ultrasound machine (ARIETTA 60, Hitachi, Chiba, Japan), measurements were taken with the 7.5 MHz linear array transducer placed in the 9th or 10th intercostal space between anterior and midaxillary lines in the zone of apposition. The participants were placed in the supine position for the measurement [11]. Only one focus-level measurement was chosen, and the depth was at diaphragm level. MDT was measured using IMT method both at total lung capacity (TLC) and at functional residual capacity (FRC) [12]. The mean IMT method is often used for measuring plaques automatically in the carotid artery. We calculated a mean of three points using IMT software: the center of the diaphragm thickness, and two surrounding points on each side (1 cm from the center of the diaphragm thickness) (Fig. 1).

Subsequently, we calculated the change ratio of diaphragm thickness (expressed in percentage) using the following formula: MDT at TLC minus MDT at FRC divided MDT at TLC [(MDTtlc—MDTfrc)/MDTtlc] × 100.

### Measurement of pulmonary function test and maximal respiratory pressure

A multifunctional spirometer (Autospiro AS507, Minato Medical Science, Osaka, Japan) was used to evaluate respiratory function. Before recording respiratory pressure data, the spirometric parameters of forced vital capacity (FVC) (%) and forced expiratory volume in one second (FEV_1_)/FVC (%) were evaluated with participants in the sitting position. Respiratory pressure examinations, as well as the spirometric examination, were also conducted with the participants in the sitting position. First, to measure exhaled air from the participant’s mouth, they had to hold a mouthpiece between their teeth. Second, they held the cylinder between their teeth using light pressure, and a clip was placed on their nose to prevent air leaking out during the respiratory tests. Finally, we recorded maximal respiratory pressure to assess respiratory muscles strength. We measured maximum expiratory pressure (MEP) or maximum inspiratory pressure (MIP) by asking participants to breath in/out as deeply as they could and breath out/in sharply and quickly. The MEP and MIP were recorded three times, and maximal end-expiratory and end-inspiratory potential data were chosen for further analysis.

### Statistical analysis

We compared the age, weight, height, BMI, BSA, mean diaphragm thickness, change ratio of the mean diaphragm thickness, and maximal end-expiratory and end-inspiratory potentials between males and females. We used the Welch t test for these analyses. Analyses were performed using GraphPad 6 (GraphPad Software, La Jolla, CA, USA). Pearson’s correlation coefficient was used to assess correlation between diaphragm measurements and the maximal respiratory potentials or anthropometric measurements. A p value < 0.05 was considered statistically significant. Moreover, we analyzed intraobserver and interobserver variabilities in the change ratio of diaphragm thickness. The intraobserver variability was assessed by estimation of the intraclass correlation coefficient using the 11 observations in the same subject by the 1st observer. Interobserver variability was tested between observations made by the 1st and 2nd observers in the same subjects.

## Results

### Intraobserver and interobserver variability

The intraobserver correlation coefficient of the change ratio in diaphragm thickness in observer 1 was 0.856 (95% confidence interval [CI] 0.492–0.961) with a p value of 0.002. The interobserver correlation coefficient of the change ratio in diaphragm thickness between the 1st and 2nd observers was 0.736 (95% CI 0.079–0.928) with a p value of 0.023.

### Baseline characters

Participant baseline characteristics by sex are shown in Table 1. Sixteen participants (57%) were female, with a mean age of 24 (interquartile range [IQR] 19–34) years, mean weight of 51 (IQR 41–58) kg, and mean height of 158 (IQR 151–165) cm. The mean BMI of the female participants was 20 (IQR 18–24) kg/m^2^. The height, weight, and BMI of males were higher than those of females. There were no significant differences with respect to sex in age, vital capacity, and FEV_1_.

**Table 1:** Baseline characteristics of 29 subjects

Variable Mean ± SD	Male (n = 13)	Female (n = 16)	p value
Age (years)	22.9 ± 3.8	23.7 ± 4.9	0.60
Body weight (kg)	64.2 ± 9.1	50.5 ± 4.6	< 0.001
Height (cm)	174.0 ± 4.5	157.8 ± 4.6	< 0.001
BMI (kg/m^2^)	21.1 ± 2.3	20.3 ± 1.7	0.29
Body surface area (m^2^)	1.7 ± 0.13	1.5 ± 0.1	< 0.001
FVC (%)	97.6 ± 9.9	98.8 ± 10.5	0.78
FVC/FEV_1_ (%)	88.6 ± 4.9	89.2 ± 5.8	0.74

FVC, forced vital capacity; FEV_1_, forced expiratory volume in one second; SD, standard deviation

### Diaphragm evaluation using ultrasonography

The MDT at FRC, MDT at TLC, and change ratio of diaphragm thickness are listed in Table 2. The MDTs at FRC and TLC in the male participants were 1.2 (IQR 0.6–1.5) and 3.8 (IQR 2.5–5.5), respectively. The MDTs at FRC and TLC in female participants were 1.5 (IQR 1.3–2.1) and 3.7 (IQR 2.3–5.2), respectively. The mean change ratio of the diaphragm thickness was 67.2% (IQR 58.2–77.6) in males and 57.2% (IQR 40.0–72.1) in females. Although the MDT at FRC in males was significantly narrower than in females (1.2 ± 0.3 mm vs 1.5 ± 0.2, p < 0.001), the change ratio of diaphragm thickness in males was larger than that in females (67.2 ± 6.2% vs 57.2 ± 7.8%, p < 0.001). There were no significant differences in the MDT at TLC with respect to sex.

**Table 2:** Ultr﻿﻿asound values of right diaphragm thickness

Variable (Mean ± SD)	Male (n = 13)	Female (n = 16)	p value
Diaphragm thickness at functional residual capacity (mm)	1.2 ± 0.3	1.5 ± 0.2	< 0.001
Diaphragm thickness at total lung capacity (mm)	3.8 ± 0.8	3.7 ± 0.8	0.82
Change ratio of diaphragm thickness (%)	67.2 ± 6.2	57.2 ± 7.8	< 0.001

### Respiratory muscle strength evaluation

Table 3 shows the mean maximal respiratory pressures in male and female participants. The mean MEPs were 88.2 (IQR 49.7–113.6) cmH_2_O and 56.8 (IQR 32.3–87.0) cmH_2_O and mean MIPs were 79.0 (IQR 44.9–116.1) cmH_2_O and 81.1 (IQR 43.5–129.6) cmH_2_O in male and female participants, respectively. While there were no significant differences in MIPs, the MEPs were higher in males than in females.

**Table 3:** Maximal respiratory pressures

Variable (Mean ± SD)	Male (n = 13)	Female (n = 16)	p value
Maximal expiratory pressure (cmH_2_O)	88.2 ± 22.5	56.8 ± 16.2	< 0.001
Maximal inspiratory pressure (cmH_2_O)	79.0 ± 23.8	81.1 ± 26.0	0.81

SD, standard deviation

### Association between the change ratio of diaphragm thickness and anthropometric data or pulmonary function test

**Fig. 2:**
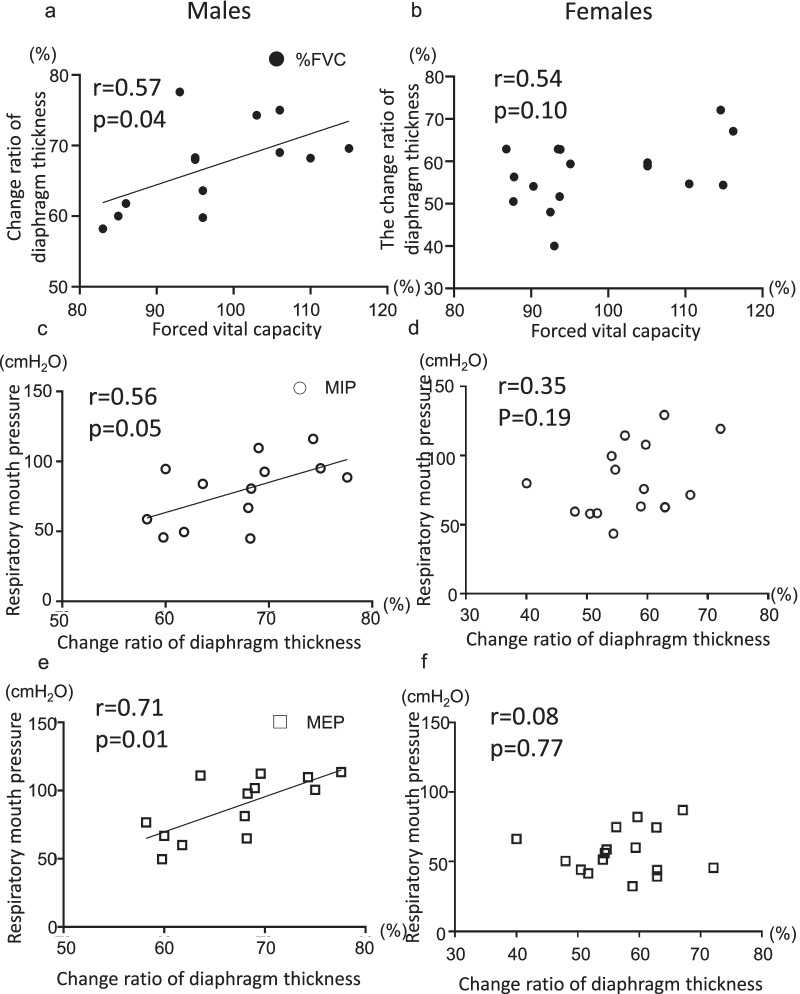
Correlation between the change ratio of diaphragm thickness and other measures in males and females. Measures included % forced vital capacity, maximum inspiratory pressure (MIP) or maximum expiratory pressure (MEP) for males (**a**, **c**, **e**), and females (**b**, **d**, **f**). There was a significant positive correlation between the change ratio of diaphragm thickness and % forced vital capacity or respiratory mouth pressures in males, but not in females

Subsequently, we assessed the correlation between the change ratio of diaphragm thickness and anthropometric data or pulmonary function test or maximal respiratory mouth pressure. Significant positive correlations were found only in males between the change ratio of diaphragm thickness and FVC (r = 0.57, p = 0.04), the change ratio of the diaphragm thickness and MIP (r = 0.56, p = 0.05), and the change ratio of the diaphragm thickness and MEP (r = 0.71, p = 0.007) (Fig. 2). Based on Fig. 2, the change ratio of the diaphragm thickness seems to be associated with FVC (r = 0.54, p = 0.10) and MIP (r = 0.35, p = 0.19).

## Discussion

In this study, we determined normal reference values for diaphragm thickness in healthy young adults with ultrasonography using the IMT method to assess respiratory muscle strength; additionally, we analyzed the relationship between diaphragm thickness and maximal respiratory mouth pressure by classifying the participants by sex. Our study revealed a positive correlation between the change ratio of diaphragm thickness and respiratory muscle strength in young adult.

A diaphragm measurement methodology using the IMT method has never been reported. Several previous reports without the IMT method show that ultrasonography measurements of diaphragm thickness have a high degree of reproducibility. In the study by Dhungana et al. [1], the coefficient of reproducibility in DMT at FRC was high (0.986 and 0.987 for intra-observer and interobserver variabilities, respectively). Although our coefficient of reproducibility in the change ratio of diaphragm thickness using the IMT method showed significant reproducibility, the coefficient value in our study was lower than that in a previous report. Dhungana et al. [1] reported that diaphragm thickness was measured by observers after an initial training of three sessions, each lesson lasting 30 min. It seemed that the coefficient of reproducibility in the previous study could be augmented with more training sessions and lesson time when compared to that in this study.

Previous studies have demonstrated various methods to observe diaphragm thickness with ultrasonography [1, 13]. Regarding the studies on diaphragm thickness with ultrasonography, Boon et al. reported a lower limit of normal diaphragm thickness with ultrasonography placed over one of the most caudal intercostal spaces of 1.4 mm in participants between 33 and 84 years of age [3]. De Bruin et al., using ultrasonography to place the right hemidiaphragm at the apposition zone, reported a mean normal diaphragm thickness at TLC for participants aged 7–12 years of 3.5 mm [14]. Our study revealed that the lower limit of the diaphragm thickness at FRC in males and females was 1.2 mm and 1.5 mm, respectively. Additionally, the mean normal diaphragm thickness at TLC in males was 3.8 mm. These results were approximately consistent with previous studies [3, 14].

Measuring respiratory mouth pressure can contribute to the assessment of respiratory muscle strength in neuromuscular disease. The mean respiratory muscle strength values have been previously reported [7, 15, 16]. The mean MIP and MEP for participants aged > 18 years were 106 and 148 cmH_2_O in males and 73 and 93 cmH_2_O in females, respectively [16]. Gibson et al. collected data from a small group of 10 healthy Caucasian women, obtaining an MEP of 67–140 cmH_2_O and MIP of 35–95 cmH_2_O, respectively [17]. Our results are similar to these results. Additionally, Heinzmann-Filho et al., despite the lack of statistical differences, found higher respiratory muscle strength values for boys than girls [6]. Our results also showed a higher MEP in males than in females. As for MIP, no significant difference was found between the sexes. To assess the MEP in young adults, we should evaluate the measurement data according to sex.

The change ratio of the diaphragm thickness appears to be a useful indicator to evaluate morphological changes of the diaphragm and differentiation based on sex. Previous studies on diaphragm thickness with ultrasonography based on sex in healthy infants and children indicated no significant differences between males and females [18]. In an adult study, Boussuges et al. found weak correlations between diaphragmatic excursion and body weight or height [19], and significant correlations between diaphragmatic measurements and FEV_1_ [5, 20]. Additionally, there was a significant relationship between MEP and body weight [6]. In this study, a positive correlation was observed between the change ratio of diaphragm thickness and respiratory muscle strength and/or FVC in healthy young adults. This result suggests that the change ratio of the diaphragm thickness correlates with breathing effort. One reason for why there was no significant positive correlation between the change ratio and respiratory muscle strength may be because of the small number of females, which may have affected the statistical analysis of the results.

Our study has some limitations. First, the sample size was small, and we only assessed young adults. A larger sample size with various age groups is necessary to draw more definitive conclusions. Second, a diaphragm measurement methodology using the IMT method has never been reported; therefore, we could not compare the diaphragm thickness results using the IMT method with previous data. Further studies are required to determine the optimal diaphragm assessment method with ultrasonography.

## Conclusions

In conclusion, the result of our study indicated that the measurement of the change ratio of diaphragm thickness using the IMT method can be accurately performed with a high degree of reproducibility. Normal reference values based on sex for diaphragm thickness and change ratio of the diaphragm thickness, evaluated by B-mode sonography in healthy young adults, were determined in this study. A positive correlation was found between the change ratio of diaphragm thickness and respiratory muscle strength in young adults; thus, the change ratio of diaphragm thickness using the IMT method may be a useful indicator for evaluating diaphragm muscle strength.

## Data Availability

The datasets used and/or analyzed during the current study are available from the corresponding author upon reasonable request.

## Abbreviations

BMI: Body mass index
BSA: Body surface area
FEV: Forced expiratory volume
FRC: Functional residual capacity
FVC: Forced vital capacity
IMT: Intima media thickness
IQR: Interquartile range
MDT: Mean diaphragm thickness
MEP: Maximum expiratory pressure
MIP: Maximum inspiratory pressure
TLC: Total lung capacity
